# Analysis of common *PTPN1 *gene variants in type 2 diabetes, obesity and associated phenotypes in the French population

**DOI:** 10.1186/1471-2350-7-44

**Published:** 2006-05-05

**Authors:** Claire Cheyssac, Cécile Lecoeur, Aurélie Dechaume, Amina Bibi, Guillaume Charpentier, Beverley Balkau, Michel Marre, Philippe Froguel, Fernando Gibson, Martine Vaxillaire

**Affiliations:** 1 CNRS UMR 8090, Institut de Biologie de Lille – Institut Pasteur, 59019 Lille, France; 2 Imperial College Genome Centre and Section of Genomic Medicine, Hammersmith campus, Imperial College, W2 0NN London, UK; 3 Service de diabétologie, Centre Hospitalier Sud-Francilien, 91106 Corbeil-Essonnes, France; 4 INSERM U780-IFR69, Research in Epidemiology and Biostatistics, 94807 Villejuif, France; 5 INSERM U695, Faculté de Médecine Xavier Bichat, Paris, France

## Abstract

**Background:**

The protein tyrosine phosphatase-1B, a negative regulator for insulin and leptin signalling, potentially modulates glucose and energy homeostasis. PTP1B is encoded by the *PTPN1 *gene located on chromosome 20q13 showing linkage with type 2 diabetes (T2D) in several populations. *PTPN1 *gene variants have been inconsistently associated with T2D, and the aim of our study was to investigate the effect of *PTPN1 *genetic variations on the risk of T2D, obesity and on the variability of metabolic phenotypes in the French population.

**Methods:**

Fourteen single nucleotide polymorphisms (SNPs) spanning the *PTPN1 *locus were selected from previous association reports and from HapMap linkage disequilibrium data. SNPs were evaluated for association with T2D in two case-control groups with 1227 cases and 1047 controls. Association with moderate and severe obesity was also tested in a case-control study design. Association with metabolic traits was evaluated in 736 normoglycaemic, non-obese subjects from a general population. Five SNPs showing a trend towards association with T2D, obesity or metabolic parameters were investigated for familial association.

**Results:**

From 14 SNPs investigated, only SNP rs914458, located 10 kb downstream of the *PTPN1 *gene significantly associated with T2D (p = 0.02 under a dominant model; OR = 1.43 [1.06–1.94]) in the combined sample set. SNP rs914458 also showed association with moderate obesity (allelic p = 0.04; OR = 1.2 [1.01–1.43]). When testing for association with metabolic traits, two strongly correlated SNPs, rs941798 and rs2426159, present multiple consistent associations. SNP rs2426159 exhibited evidence of association under a dominant model with glucose homeostasis related traits (p = 0.04 for fasting insulin and HOMA-B) and with lipid markers (0.02 = p = 0.04). Moreover, risk allele homozygotes for this SNP had an increased systolic blood pressure (p = 0.03). No preferential transmission of alleles was observed for the SNPs tested in the family sample.

**Conclusion:**

In our study, *PTPN1 *variants showed moderate association with T2D and obesity. However, consistent associations with metabolic variables reflecting insulin resistance and dyslipidemia are found for two intronic SNPs as previously reported. Thus, our data indicate that *PTPN1 *variants may modulate the lipid profile, thereby influencing susceptibility to metabolic disease.

## Background

The ubiquitously expressed protein tyrosine phosphatase-1B (PTP1B), encoded by the *PTPN1 *gene, catalyzes the dephosphorylation of tyrosine residues from the insulin receptor kinase activation segment [1] and IRS1 [2] resulting in the down-regulation of insulin signalling. PTP1B also inhibits leptin signalling through the dephosphorylation of JAK2 and STAT3 [3,4]. The disruption of the *PTPN1 *gene in mice results in increased insulin sensitivity, resistance to diet-induced obesity [5] and enables normalization of blood glucose levels [6]. Moreover, it was shown that the inactivation of *PTPN1 *with antisense oligonucleotides regulates the expression of genes involved in lipogenesis, such as *SREBF1*, suggesting that PTP1B may play a role in the enlargement of adipocyte energy storage [7]. Taken together, these data illustrate a crucial role for PTP1B in insulin and leptin pathways and suggest that abnormal PTP1B activity could lead to insulin resistance and thereby to T2D and to obesity.

The human *PTPN1 *gene maps on chromosome 20q13.13, a syntenic region of the distal arm of the murine chromosome 2 that harbours quantitative trait loci for body fat and body weight [8]. In humans, several linkage signals with T2D [9], BMI [10], fat mass and energetic intake [8,11,12] were reported at this locus in different populations, further supporting *PTPN1 *candidacy in T2D and obesity. This locus also showed evidence of linkage with early onset T2D (onset = 45 years) in a subset of 55 French families [13]. Several recent studies have investigated genetic variants of *PTPN1 *for association with T2D. In an extensive analysis of the *PTPN1 *gene locus, Bento et al. [14] found convincing associations between multiple SNPs and T2D in two independent Caucasian American case-control samples. All of the associated SNPs were found to lie in a single 100 kb haplotype block and one common haplotype (frequency = 36%) was found to be strongly associated with T2D. The same group evaluated SNPs and haplotypes of *PTPN1 *for association with quantitative glycaemic traits in Hispanic American subjects from the Insulin Resistance Atherosclerosis Study Family Study (IRASFS) [15]. Again, multiple SNPs were found to be associated with the insulin sensitivity index (Si) and fasting glucose. Haplotypes that were previously associated with T2D risk also presented association with lower Si and with higher fasting glucose in the IRAS family study. However, a recent meta-analysis including 7883 individuals from three large European case-control samples (from US, Poland and Scandinavia) did not replicate this association for any single SNP or haplotype [16]. These divergent findings question the impact of variation in the *PTPN1 *gene on the risk of T2D in populations of different ethnic origin. The aim of the present study was to further investigate the contribution of common *PTPN1 *polymorphisms to the risk of T2D and obesity, and to the variability of quantitative metabolic traits in the French population.

## Methods

### Subjects

Two independently ascertained case-control samples were used to investigate association between *PTPN1 *gene variants and T2D. The first case group (D1) is composed of 325 unrelated T2D probands from French families with strong T2D aggregation recruited at the CNRS unit in Lille [13]. The D1 probands were compared to a group of 311 unrelated normoglycaemic subjects called C1 (age> 45 years; fasting glycaemia< 5.6 mM) selected among spouses of French families recruited at the CNRS unit in Lille. The second case group, called D2, consists of 902 diabetes subjects recruited at the Endocrinology-Diabetology Department of the Corbeil-Essonnes Hospital. The D2 diabetes subjects were compared to a second group of 736 normoglycaemic (fasting glycaemia< 5.6 mM), non-obese subjects (C2) selected from the D.E.S.I.R. cohort [17]. To assess the association between SNPs and obesity, two groups of obese subjects were studied: a group of 616 subjects with moderate obesity (30 kg/m^2^< BMI< 40 kg/m^2^) referred to as OBM and a group of 688 subjects with severe obesity (BMI> 40 kg/m^2^) termed SO. Both groups of obese subjects were compared to the C2 control group. The clinical characteristics of the case and control groups are given in Table 1. Familial association tests were performed in 148 French Caucasian nuclear families and in a subset of 55 sib-pairs characterized by an early age-of-onset of T2D (before 45 years) previously presenting linkage with T2D [13]. The entire family sample set include a total of 633 individuals, of whom 432 presented with diabetes (sex ratio: 198/234, mean age-at-diagnosis: 49.5 ± 10.6 years, mean BMI: 27.9 ± 4.5 kg/m^2^), 72 with glucose intolerance (sex ratio: 36/36, mean age-at-diagnosis: 59.1 ± 9.6 years, mean BMI: 27.4 ± 4.7 kg/m^2^), and 129 were normoglycaemic subjects (sex ratio: 40/89, mean BMI: 25.1 ± 4.1 kg/m^2^).

**Table 1:** Characteristics of the case and control groups studied

Populations	n	Sex ratio men/women	Mean age-at-diagnosis (years)	Mean age-at-examination (years)	Mean BMI (kg/m^2^)
Diabetic subjects recruited in Lille (D1)	325	175/150	45.50 ± 10.71	61.83 ± 10.51	26.64 ± 3.63
Diabetic subjects recruited in Corbeil-Essonnes hospital (D2)	902	518/384	51.76 ± 8.87	62.55 ± 9.50	30.46 ± 6.02
Subjects with moderate obesity (OBM)	616	275/341	-	50.11 ± 14.15	34.21 ± 3.61
Subjects with severe obesity (SO)	688	165/523	-	46.03 ± 11.67	47.55 ± 7.33
Control subjects (C1)	311	123/188	-	62.99 ± 10.93	25.80 ± 4.57
Control subjects from D.E.S.I.R. cohort (C2)	736	293/443	-	53.47 ± 5.65	23.25 ± 1.78

For each group the number of subjects (n) and the sex ratio (men/women) are given. The mean age-at-diagnosis is the age of the patient when diabetes was diagnosed. The mean age-at-diagnosis, the mean age and the mean BMI are given as means ± standard deviation.

### Genotyping

SNPs were genotyped using the Applied Biosystem SNPlex™ Technology based on the Oligonucleotide Ligation Assay (OLA) combined with multiplex PCR, to achieve target amplification and allelic discrimination [18]. This allows multiplex genotyping for 48 SNPs simultaneously in a unique sample. Allelic discrimination is performed through a capillary electrophoresis analysis using an Applied Biosystems 3730xl DNA Analyzer and the GeneMapper3.7 software. Thirty two individuals were genotyped in duplicate to assess the genotyping accuracy. All SNPs gave a genotyping concordance rate of 100%. SNPs rs941798 and rs914458 were genotyped in the family sample set by direct sequencing using an automated ABI Prism 3700 DNA sequencer in combination with the Big Dye Terminator Cycle Sequencing Ready Reaction Kit (Applied Biosystems, Foster City, California, United States). For SNP-7077G/C, genotyping in the families was performed by the FRET technology using the LightCycler™ assay based on hybridization probes labelled with fluorescent dyes (Roche Diagnostics, Basel, Switzerland). In order to assess genotyping accuracy for this SNP, more than 10% of the genotypes were checked by direct sequencing and presented a concordance rate in excess of 99%.

### Tag SNP selection

The genotypes of 29 SNPs in 30 Caucasian European trios available from the HapMap database (version of January 2005) were analysed using the Haploview software, and seven tag SNPs were selected according to the pairwise linkage disequilibrium calculated using the four gamete rule.

### Statistical analysis

The Chi-square test was used to assess the deviation of SNP genotypes from the Hardy-Weinberg Equilibrium (HWE) and to examine the association of SNPs with T2D and obesity [19]. Significant association was considered for a p-value< 0.05. The Mantel-Haenzsel interaction test was used to define odds-ratios for the combined case-control analysis [20]. Four quantitative traits related to glucose homeostasis were analysed in the control group C2: fasting glucose (Go expressed in mM); fasting insulin (Io expressed in μU/ml); HOMA-B, defined by the formula HOMA-B = (Io*20)/(Go-3.5); and HOMA-IR, defined by the formula HOMA-IR = (Io*Go)/22.5. BMI (kg/m^2^), total cholesterol (mM), HDL cholesterol (mM), LDL cholesterol (mM), triglycerides (mM), systolic and diastolic blood pressure were also analysed in this control group. Non-Gaussian variables were either log or square-root transformed. Linear regressions were then performed to adjust each variable for covariates such as age, sex and BMI. According to the normality of the standardized residuals, ANOVA or non-parametric tests were performed and three genetic models (codominant, dominant and recessive) were investigated. Linear regressions, ANOVA and non-parametric tests were performed using the SPSS statistical analysis software. Familial association was investigated using the Family Based Association Test (FBAT) [21]. The Haploview software [22] was used to determine the pairwise linkage disequilibrium (D') and the minimal set of SNPs displaying the genetic information. This was checked with the STRATEGY software [23]. Haplotype analyses were then performed using the Haplotype Trend Regression (HTR) programme [24] to test haplotypic association with T2D, obesity status and adjusted quantitative variables. The relevance of the best haplotypic association was computed using 1000 permutations and the Cocaphase software [25]. The statistical power of our combined sample of diabetes and control subjects was evaluated through the Quanto software [26] using the log-additive model of inheritance. Prevalence of T2D was estimated at 5%, and power was calculated for 1.2= OR= 1.4.

## Results

**Figure 1:**
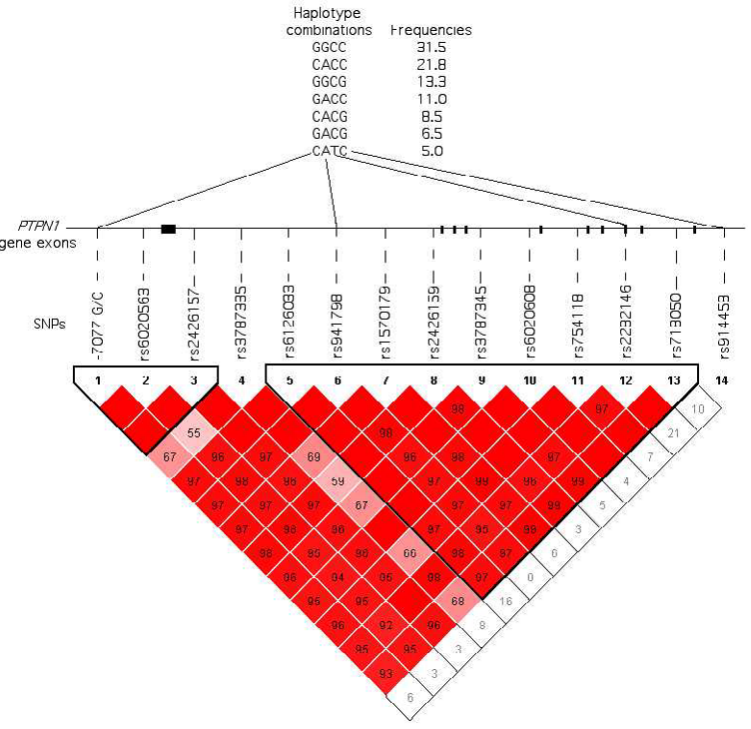
Linkage disequilibrium plot of the *PTPN1 *gene region evaluated in a group of 736 French normoglycaemic non-obese subjects The *PTPN1 *gene is shown by the black line with boxes representing its 10 exons. The localisation of the selected SNPs is indicated by the dotted line. The linkage disequilibrium plot represents the pairwise LD (D') estimated from the control group (C2) using the four gamete rule implemented in the Haploview software. The values indicated in each square is the D' value, when no value is indicated D' = 1. The red colour indicates strong (D'> 0.8) and statistically significant (LOD>2) LD. Above the gene, the haplotype combinations are indicated with their respective frequencies; they were determined using the Haploview and Strategy softwares.

Fourteen *PTPN1 *SNPs were selected for the study, including: (i) six common *PTPN1 *gene promoter or intronic SNPs (-7077G/C, rs941798, rs1570179, rs3787345, rs754118 and rs718050) showing association with T2D in previous studies [14,15,27]; (ii) seven tag SNPs selected from the HapMap version 14 of January 2005; and (iii) the synonymous exon 8 variant rs2282146, previously referred to as P303P [28]. The linkage disequilibrium (LD) structure at the *PTPN1 *locus was evaluated from the fourteen SNPs genotyped in a group of 736 French normoglycaemic non-obese subjects (C2). As shown in Figure 1, the LD analysis defined two strongly correlated haplotype blocks (D' = 0.98). Moreover the LD matrix showed high pairwise LD between 12 of the 14 SNPs genotyped. The SNP rs3787335 presented a moderate LD (0.55=D'= 0.70) with 7 SNPs leading to a two block structure covering ~91 kb at the *PTPN1 *locus and SNP rs914458, located 10 kb downstream of *PTPN1*, that showed no LD with the other 13 SNPs genotyped. Thus, our data indicated that *PTPN1 *lies in a region of strong LD, in agreement with previous reports [14,16].

To assess the influence of the *PTPN1 *genetic variation on T2D and obesity risk in the French population, the 14 common SNPs (minor allele frequency (MAF) >5%) were genotyped in two case-control cohorts described in Table 1. Only SNP rs914458 deviated from the Hardy-Weinberg equilibrium (HWE) in the group of subjects presenting with a severe obesity (p = 0.005). To avoid genotyping errors, 17% of the total genotypes were verified by direct sequencing, resulting in a 99.1% concordance rate.

In our initial T2D association analysis, we compared the allelic frequencies of the 14 SNPs between 325 probands from T2D French families (D1) and a group of 311 normoglycaemic controls (C1). As shown in Table 2, only SNP rs6020563 presented a nominal allelic association with T2D (p = 0.04, OR = 1.29 [1.01–1.65]). This association is slightly stronger under a recessive model (p = 0.031, OR = 1.52 [1.04–2.23]; data not shown). In our second independently ascertained case-control study comprising 902 French adult diabetes subjects (D2) and 736 normoglycaemic subjects from the general population (C2), none of the 14 SNPs tested were associated with T2D. In order to assess a possible confounding role of BMI on the diabetes status, the D2 diabetes subjects were divided into two sub-groups: (i) subjects with BMI< 27 kg/m^2 ^(n = 277); and (ii) subjects with BMI= 27 kg/m^2 ^(n = 625). Neither of the sub-groups showed evidence of association with T2D (data not shown). To enhance statistical power, combined odds-ratios were then determined for each SNP by the Mantel-Haenszel test, except for rs6020563 which showed a significant difference in allelic frequencies between the two case-control studies (p = 0.019). In the combined sample, no significant differences of allele frequency were observed for the 13 remaining SNPs, as shown in Table 2. However, SNP rs914458, located 10 kb downstream of *PTPN1*, associated with T2D when analysed under a dominant model (p = 0.02; Combined OR = 1.43 [1.06–1.94]). In order to assess the haplotypic effects, the Haploview and Strategy software were used to define a minimal set of 4 SNPs (-7077 G/C, rs941798, rs2282146 and rs914458), which were identified as tagging the seven common haplotypes (frequency= 5%), and accounted for 97.6% of the haplotypic diversity (Figure 1). In the combined sample, haplotype CACG, including the G "protective" allele of the associated SNP rs914458, was more frequent in the control subjects than in the diabetes cases (Table 3) suggesting a potential "protective" effect. In order to better estimate the significance of this effect, we computed the p-value for the association in 1000 permutations of the dataset. The empirical p-value obtained (p = 0.22) was not statistically significant, suggesting that the association could have been observed by chance.

**Table 2:** Type 2 Diabetes case-control association study of the 14 *PTPN1 *SNPs.

	First T2D case-control study				Second T2D case-control study				All cases vs all controls				
	
	MAF in group D1 (n = 325)	MAF in group C1 (n = 311)	Allelic p-values	OR [95% CI]	MAF in group D2 (n = 902)	MAF in group C2 (n = 736)	Allelic p-values	OR [95% CI]	MAF in the total group of cases (n = 1227)	MAF in the total group of controls (n = 1047)	Allelic p-values	Dominant model p-values	Recessive model p-values
-7077 G/C	31.7	36.3	0.09	1.23 [0.97–1.55]	36.6	37.4	0.63	1.04 [0.90–1.20]	35.3	37.1	0.20	0.17	0.43
rs6020563 T/G	44.0	50.4	**0.04**	**1.29 [1.01–1.65]**	49.3	47.3	0.26	1.09 [0.94–1.26]	48.0	48.1	-	-	-
rs2426157 A/G	25.5	26.6	0.66	1.06 [0.82–1.36]	28.0	29.0	0.53	1.05 [0.90–1.23]	27.3	28.3	0.44	0.14	0.92
rs3787335 T/G	6.3	8.5	0.14	1.38 [0.90–2.12]	7.7	8.1	0.73	1.05 [0.81–1.35]	7.4	8.2	0.29	0.85	0.26
rs6126033 C/T	5.4	7.5	0.12	1.43 [0.90–2.26]	6.5	6.8	0.80	1.04 [0.78–1.38]	6.2	7.0	0.30	0.85	0.30
rs941798 A/G	51.4	47.5	0.17	1.17 [0.94–1.46]	46.4	45.1	0.48	1.05 [0.91–1.21]	47.8	45.8	0.18	0.33	0.27
rs1570179 C/T	31.8	34.4	0.34	1.12 [0.89–1.42]	34.7	35.4	0.65	1.03 [0.89–1.20]	33.9	35.1	0.38	0.17	0.80
rs2426159 A/G	43.6	48.2	0.10	1.20 [0.96–1.50]	46.2	47.2	0.59	1.04 [0.90–1.20]	45.5	47.5	0.18	0.56	0.13
rs3787345 C/T	36.0	40.7	0.09	1.22 [0.97–1.53]	40.6	39.8	0.66	1.03 [0.89–1.19]	39.2	40.0	0.58	0.46	0.84
rs6020608 C/T	27.1	27.7	0.82	1.03 [0.80–1.32]	28.5	29.1	0.75	1.03 [0.88–1.20]	28.1	28.7	0.70	0.20	0.83
rs754118 C/T	33.6	35.1	0.57	1.07 [0.84–1.36]	34.9	35.6	0.67	1.03 [0.89–1.20]	34.5	35.5	0.51	0.15	0.94
rs2282146 C/T	5.9	6.7	0.55	1.15 [0.72–1.82]	6.0	6.7	0.47	1.11 [0.84–1.48]	6.0	6.7	0.35	0.91	0.34
rs718050 G/A	34.4	37.0	0.36	1.12 [0.88–1.41]	36.8	36.3	0.80	1.02 [0.88–1.18]	36.1	36.5	0.78	0.79	0.85
rs914458 C/G	27.5	30.5	0.26	1.16 [0.90–1.49]	29.1	30.4	0.42	1.07 [0.91–1.25]	28.7	30.4	0.20	**0.02**	0.78

All SNPs were in Hardy-Weinberg equilibrium in the different groups tested. Minor Allele Frequencies (MAF) are given for each group and for the pooled sample. P-values, odds-ratios (OR) and 95% confidence interval (95%CI) were estimated by a chi-square test for both initial case-control studies. Individual ORs are given for each SNP as "at-risk" vs. "protective" alleles. The combined p-values were determined by the Mantel-Haenszel interaction test. For the combined analysis, the p-values for dominant and recessive genetic models are also given. For SNP rs914458, which showed an association under the dominant model from the combined analysis, the OR value was 1.43, [95%CI: 1.06–1.94].

**Table 3:** Type 2 Diabetes association analysis for the seven common haplotypes

Haplotypes	Frequencies				Frequencies				Frequencies			
									
	D1	C1	Individual p-values	Overall p-value	D2	C2	Individual p-values	Overall p-value	D1 + D2 cases	C1 + C2 controls	Individual p-values	Overall p-value
GGCC	37.8	34.3	0.13		31.9	31.5	0.59		33.5	32.3	0.23	
CACC	18.3	19.9	0.42		24.0	21.8	0.40		22.4	21.1	0.67	
GGCG	13.8	12.2	0.61		13.8	13.3	0.97		13.7	13.0	0.88	
GACC	11.8	11.7	0.93	0.44	10.7	11.0	0.97	0.42	11.0	11.3	0.95	0.20
CACG	7.1	9.1	0.17		6.1	8.5	0.07		6.4	8.7	**0.02**	
GACG	5.1	5.4	0.78		6.6	6.5	0.85		6.2	6.1	0.87	
CATC	3.9	3.4	0.96		4.2	5.0	0.45		4.2	4.6	0.48	

The 7 haplotypes defined by the four most informative SNPs (-7077 G/C, rs941798, rs2282146 and rs914458) were tested for association with T2D in both the individual case-control samples and in the pooled sample. Frequencies were estimated from case and control groups separately using the HTR software. Individual and overall p-values were indicated. We estimated the empirical p-value of the CACG haplotype in the combined sample through 1000 permutations of the dataset using the Cocaphase software (p = 0.20).

We also investigated the potential contribution of the 14 common *PTPN1 *SNPs to obesity risk. SNPs were genotyped in a group of 616 subjects presenting with moderate obesity (30 kg/m^2^<BMI< 40 kg/m^2^) and a group of 688 subjects presenting with severe obesity (BMI = 40 kg/m^2^). Both groups were compared to the C2 group of 736 non diabetic and non-obese control subjects. SNP rs914458 displayed an association with moderate obesity (p = 0.04, OR = 1.2 [1.01–1.43], Table 4) and a trend towards association with morbid obesity. The effect of the haplotypes was also investigated in both groups of obese subjects. In spite of a non-significant overall p-value, the CACG haplotype was significantly more frequent in the control group compared to the moderately obese subjects group (Table 4). However, the empirical p-value estimated through permutation testing, was not significant (p = 0.30) suggesting that the association observed with moderate obesity was probably due to chance.

**Table 4:** Obesity case-control association study of the 14 *PTPN1 *SNPs and haplotypes.

				OBM vs C2		SO vs C2	
				
	MAF in the OBM group (n = 616)	MAF in the SO group (n = 688)	MAF in the C2 group (n = 736)	Allelic p-values	OR [95% CI]	Allelic p-values	OR [95% CI]
-7077 G/C	38.4	38.0	37.4	0.60	1.04 [0.89–1.22]	0.83	1.02 [0.87–1.19]
rs6020563 T/G	48.4	50.2	47.3	0.56	1.05 [0.89–1.23]	0.14	1.12 [0.96–1.31]
rs2426157 A/G	28.9	27.8	29.0	0.96	1.00 [0.84–1.18]	0.39	1.07 [0. 91–1.27]
rs3787335 T/G	8.5	9.6	8.1	0.67	1.06 [0.81–1.40]	0.13	1.22 [0.94–1.58]
rs6126033 C/T	7.0	8.7	6.8	0.81	1.04 [0.77–1.41]	**0.05**	1.32 [1.0–1.75]
rs941798 A/G	44.6	45.2	45.1	0.76	1.02 [0.88–1.19]	0.97	1.00 [0.86–1.16]
rs1570179 C/T	35.4	35.9	35.4	0.99	1.00 [0.85–1.17]	0.82	1.02 [0.87–1.19]
rs2426159 A/G	48.9	49.4	47.2	0.36	1.07 [0.92–1.25]	0.22	1.10 [0.95–1.27]
rs3787345 C/T	40.4	42.0	39.8	0.76	1.02 [0.88–1.20]	0.23	1.10 [0.94–1.28]
rs6020608 C/T	28.7	27.8	29.1	0.85	1.02 [0.86–1.20]	0.41	1.07 [0. 91–1.12]
rs754118 C/T	35.5	37.4	35.6	0.93	1.00 [0.86–1.18]	0.35	1.08 [0.92–1.27]
rs2282146 C/T	6.2	7.0	6.7	0.65	1.08 [0.77–1.53]	0.69	1.06 [0.79–1.43]
rs718050 G/A	36.7	38.7	36.3	0.84	1.02 [0.87–1.19]	0.20	1.11 [0.95–1.29]
rs914458 C/G	26.7	27.5	30.4	**0.04**	1.20 [1.01–1.43]	0.07	1.17 [0.99–1.39]

Haplotype	Frequencies in OBM	Frequencies in SO	Frequencies in C2	Individual p-values	Overall p-value	Individual p-values	Overall p-value

GGCC	32.3	34.4	31.5	0.53		0.17	
CACC	24.3	20.5	21.8	0.25		0.71	
GGCG	12.0	11.3	13.3	0.22		0.19	
GACC	12.2	11.1	11.0	0.38	0.32	0.91	0.78
CACG	5.8	8.7	8.5	**0.04**		0.58	
GACG	6.2	6.3	6.5	0.58		0.70	
CATC	4.7	6.0	5.0	0.78		0.55	

Genotype and allele frequencies were estimated for each group. All genotypes were in Hardy-Weinberg equilibrium in the populations tested, except for SNP rs914458 C/G in the group of subjects with severe obesity. The p-values for chi-square tests, odds-ratios (ORs) and 95% confidence intervals (CI95) are given. The individual and overall p-values of the haplotypes are indicated. We estimated the empirical p-value of the CACG haplotype in the group of moderate obese subjects (p = 0.30) using 1000 permutations.

In order to assess the influence of the common *PTPN1 *gene variants on quantitative metabolic variables, association analyses were performed in the C2 group of normoglycaemic and non-obese subjects. Tables 5 and 6 present the results of the genotype-quantitative traits correlation studies for glucose and lipid metabolism traits, respectively. Two SNPs located in intron 1, rs941798 and rs2426159 (D' = 0.99), were strongly correlated and showed multiple associations with metabolic parameters as previously reported for SNP rs941798 [14]. SNP rs941798 displayed an association with HOMA-B in a dominant model (p = 0.03), whilst in a recessive model it was associated with decreased HDL cholesterol (p = 0.05) and increased triglyceride levels (p = 0.03). SNP rs2426159 showed associations with increased fasting insulin (p = 0.04), HOMA-B (p = 0.04), triglycerides (p = 0.02), and LDL cholesterol levels (p = 0.03), decreased HDL cholesterol level (p = 0.03) and with an increased systolic blood pressure (p = 0.03). Taken together these results suggest an association with the metabolic syndrome. No associations were observed for BMI, HOMA-IR, or total cholesterol. Finally, no *PTPN1 *haplotype was associated with any of the quantitative traits (data not shown).

**Table 5:** Analysis of associations between genotypes and phenotypes related with T2D in the C2 group.

	Genotypes	N	Go (mmol/l)*		Io (mSI/ml)		HOMA-B		HOMA-IR*	
			
			Mean	pdom prec	Mean	pdom prec	Mean	pdom prec	Mean	pdom prec
-7077G/C	GG	283	5.03 ± 0.37	0.41	5.02 ± 2.45	0.43	68.63 ± 35.42	0.14	1.13 ± 0.58	0.63
	GC	336	5.05 ± 0.35	0.37	5.24 ± 4.81	0.13	66.44 ± 32.30	**0.05**	1.19 ± 1.11	0.16
	CC	102	5.10 ± 0.34		4.57 ± 1.85		58.96 ± 25.34		1.04 ± 0.44	
rs6020563	TT	186	5.05 ± 0.34	0.83	5.23 ± 2.52	**0.05**	69.98 ± 35.11	0.07	1.18 ± 0.60	0.08
	TG	319	5.05 ± 0.36	0.98	5.17 ± 4.88	0.07	65.68 ± 31.96	0.1	1.17 ± 1.13	0.11
	GG	150	5.06 ± 0.34		4.63 ± 1.89		61.23 ± 25.65		1.05 ± 0.47	
rs941798	AA	223	5.08 ± 0.33	0.23	4.69 ± 1.93	0.15	61.23 ± 25.81	**0.03**	1.06 ± 0.46	0.31
	AG	345	5.03 ± 0.37	0.85	5.23 ± 4.88	0.06	67.75 ± 35.89	0.075	1.18 ± 1.13	0.06
	GG	153	5.05 ± 0.35		5.20 ± 2.24		70.15 ± 34.05		1.17 ± 0.54	
rs2426159	AA	202	5.04 ± 0.35	0.64	5.21 ± 2.51	**0.04**	70.36 ± 37.00	**0.05**	1.18 ± 0.60	0.07
	AG	360	5.05 ± 0.36	0.81	5.19 ± 4.71	0.06	66.71 ± 35.32	0.06	1.17 ± 1.09	0.15
	GG	161	5.06 ± 0.35		4.66 ± 2.01		61.68 ± 26.88		1.05 ± 0.45	
rs3787345	CC	261	5.03 ± 0.37	0.31	5.05 ± 2.49	0.55	69.12 ± 36.16	0.15	1.14 ± 0.59	0.82
	CT	332	5.05 ± 0.34	0.18	5.06 ± 2.44	0.115	67.28 ± 35.07	**0.03**	1.14 ± 0.57	0.21
	TT	116	5.10 ± 0.34		5.28 ± 7.38		59.37 ± 25.80		1.21 ± 1.70	
rs6020608	CC	366	5.02 ± 0.38	0.17	4.95 ± 2.36	0.79	67.93 ± 34.04	0.12	1.12 ± 0.56	0.92
	CT	291	5.06 ± 0.33	**0.03**	5.25 ± 5.08	0.79	65.54 ± 32.22	0.16	1.19 ± 1.17	0.99
	TT	64	5.15 ± 0.33		4.80 ± 2.12		59.72 ± 59.72		1.10 ± 0.50	
rs754118	CC	297	5.01 ± 0.38	**0.05**	4.97 ± 2.44	0.92	68.76 ± 35.38	0.09	1.12 ± 0.58	0.73
	CT	314	5.07 ± 0.33	0.18	5.28 ± 4.93	0.36	65.67 ± 31.93	0.14	1.20 ± 1.14	0.5
	TT	94	5.11 ± 0.34		4.71 ± 1.99		60.19 ± 26.53		1.08 ± 0.47	
rs718050	GG	290	5.01 ± 0.38	0.09	5.03 ± 2.48	0.54	69.50 ± 35.55	**0.05**	1.13 ± 0.59	0.77
	GA	324	5.06 ± 0.33	0.07	5.23 ± 4.86	0.30	65.79 ± 32.21	0.07	1.18 ± 1.13	0.48
	AA	96	5.12 ± 0.34		4.65 ± 1.90		58.93 ± 24.56		1.06 ± 0.45	

Data are means ± SD for nonadjusted variables. Variables were transformed and adjusted by covariates (age, sex, BMI). The G0 and HOMA-IR variables had non-Gaussian residuals and were thus analysed by non-parametric tests as indicated by an asterisk (*); other variables were analysed by an ANOVA test. Results were presented for dominant and recessive models. Only SNPs showing an association were presented.

**Table 6:** Association analysis with quantitative traits related to the lipid and arterial blood pressure profiles

			HDL*		LDL		Triglycerides		Systolic pressure*		Diastolic pressure*	
			
SNPs	Genotypes	N	Mean	pdom prec	Mean	pdom prec	Mean	pdom prec	Mean	pdom prec	Mean	pdom prec
rs2426157	AA	367	1.75 ± 0.44	0.12	3.65 ± 0.84	**0.05**	0.97 ± 0.51	0.68	131.3 ± 13.0	**0.03**	78.1 ± 8.4	0.87
	AG	289	1.77 ± 0.45	0.29	3.55 ± 0.80	0.52	0.98 ± 0.50	0.27	129.2 ± 12.5	**0.04**	77.9 ± 8.7	**0.02**
	GG	65	1.77 ± 0.40		3.55 ± 0.84		0.94 ± 0.51		133.6 ± 10.5		80.8 ± 7.2	
rs941798	AA	223	1.78 ± 0.42	0.06	3.54 ± 0.86	0.13	0.94 ± 0.47	0.09	130.8 ± 12.1	0.89	78.7 ± 7.8	0.53
	AG	345	1.75 ± 0.46	**0.05**	3.61 ± 0.80	0.17	0.97 ± 0.48	**0.03**	130.4 ± 13.2	0.20	78.2 ± 8.9	0.87
	GG	152	1.71 ± 0.41		3.69 ± 0.83		1.05 ± 0.60		131.6 ± 12.7		78.1 ± 8.9	
rs2426159	AA	201	1.72 ± 0.43	**0.03**	3.71 ± 0.84	**0.03**	1.04 ± 0.58	**0.02**	132.4 ± 14.2	**0.03**	78.6 ± 8.9	0.36
	AG	360	1.77 ± 0.45	0.65	3.58 ± 0.81	0.20	0.95 ± 0.47	0.38	130.1 ± 12.2	0.98	78 ± 8.7	0.82
	GG	161	1.77 ± 0.44		3.51 ± 0.83		0.94 ± 0.48		129.9 ± 11.2		78.4 ± 7.4	
rs6020608	CC	366	1.74 ± 0.44	0.18	3.65 ± 0.84	0.08	0.97 ± 0.51	0.87	131.5 ± 13.4	**0.03**	78.2 ± 8.8	0.88
	CT	291	1.77 ± 0.45	0.81	3.55 ± 0.81	0.83	0.99 ± 0.50	0.61	129.2 ± 12.2	**0.02**	77.9 ± 8.4	**0.03**
	TT	64	1.72 ± 0.35		3.58 ± 0.82		0.95 ± 0.50		133.4 ± 10.8		80.7 ± 7.5	

Data are means ± SD for nonadjusted variables. Variables were transformed and adjusted by covariates (age, sex, BMI). The HDL and blood pressure measures variables had non-Gaussian residuals and were analysed by non-parametric tests as indicated by an asterisk (*); other variables were analysed by an ANOVA test. Results were presented for dominant and recessive models. Only SNPs showing an association are presented here.

Three non redundant SNPs (-7077 G/C, rs941798 and rs914458) presenting trends or associations with T2D in the case-control study, or with quantitative traits, were further investigated in the entire family sample set comprising 148 French families and in a subset of 55 sib-pairs presenting with an early age-of-onset of T2D and having shown linkage at the 20q13 locus. No preferential transmission of alleles was observed for the SNPs tested in either of the samples (using the FBAT software; data not shown).

## Discussion

In this study, we investigated the effect of *PTPN1 *genetic variation on susceptibility to T2D and obesity, and also on quantitative metabolic parameters. Our T2D association results strongly differ from those obtained in Caucasian American populations [14] and in Hispanic Americans from IRASFS [15] but are in good accordance with the meta-analysis conducted by Florez et al. [16]. Indeed, in the meta-analysis of the French case-control samples, only an extragenic SNP, rs914458 located 10 kb downstream of the *PTPN1 *gene, showed moderate association with T2D under a dominant model (p = 0.02). These divergent findings could be due to heterogeneity of T2D aetiology among the different populations, perhaps driven by differences in genetic or environmental modifiers. Indeed, we note that the Bento et al. and Palmer et al. studies displaying associations to T2D were performed in American subjects, whereas the Florez study and the present study were focused on European populations. The hypothesis of a lack of power seems unlikely for SNPs with an "at-risk" allele frequency> 24%. According to the Quanto software, for such an allele frequency and considering ORs between 1.2 and 1.4, our combined case-control sample (including 1227 diabetic subjects and 1047 controls) provide more than 80% power to detect an association with T2D (power of 80.4% for OR = 1.2, and power of 99.9% for OR = 1.4). Thus, we expect that our study has good power to replicate the Bento et al results. However, we cannot formally exclude the possibility that our study design does not allow us to observe associations for SNPs rarer than 24% (rs3787335, rs6126033 and rs2282146).

Only a few association studies between *PTPN1 *gene variants and obesity status have been reported previously. Here, we show a weak association between SNP rs914458 and moderate obesity (p = 0.04) and a trend towards association with severe obesity for SNP rs6126033 located in the first intron (p = 0.05). However, our association analysis of metabolic syndrome quantitative traits supports the hypothesis of a possible influence of *PTPN1 *genetic variation on insulin sensitivity, plasma lipid levels and hypertension which are characteristics of the metabolic syndrome. Indeed, multiple consistent associations were observed between SNPs rs941798 and rs2426159 and metabolic parameters reflecting insulin sensitivity and the lipid profile. These results are in accordance with a number of studies showing influence of *PTPN1 *SNPs on metabolic syndrome traits. Recently, Spencer-Jones et al [29] reported several associations between *PTPN1 *gene variants and insulin sensitivity quantitative traits. Kipfer-Coudreau et al [30] showed association between *PTPN1 *genetic variation and dyslipidemia in the French population. An association was also reported between the Pro387Leu variant and hypertriglyceridemia in a German population [31]. In addition, Olivier et al. reported associations of *PTPN1 *gene variants with BMI and total cholesterol level in an Asian population [27]. These results are consistent with the known role of *PTPN1 *in the dephosphorylation of the JAK2 kinase, an essential event in the leptin signalling pathway, and in the regulation of the expression of the lipogenic *SREBF1*, *LPL *and *PPARγ *genes [7].

## Conclusion

Taken together, our data indicate that *PTPN1 *variants may modify the lipid profile, thereby influencing susceptibility to the metabolic syndrome in the French population. Further genetic and functional studies of the contribution of *PTPN1 *variation to the metabolic syndrome and related traits are clearly warranted.

## Abbreviations

T2D: type 2 diabetes

*PTPN1*: protein tyrosine phosphatase non-receptor type1

JAK2: Janus kinase 2

BMI: Body Mass Index

SNP: Single Nucleotide Polymorphisms

OLA: Oligonucleotide Ligation Assay

DNA: desoxyribonucleic acid

PCR: polymerase chain reaction

FRET: fluorescence resonnance energy transfert

HTR: Haplotype Trend Regression

FBAT: Family Based Association Test

GIST: Genotype IBD Sharing Test

NPL: non parametric linkage

HOMA-B: Homeostasis Model Assessment β-cell insulin secretory index

HOMA-IR: Homeostasis Model Assessment Insulin Resistance

HDL: high density level

LDL: low density level

ANOVA: Analysis of Variance

## Competing interests

The author(s) declare that they have no competing interests.

## Authors' contributions

Claire Cheyssac contributed to the study design and SNP selection for genotyping, to the statistical analyses and to the preparation and writing of the paper. Cecile Lecoeur performed the familial analysis (FBAT and TDT). Aurelie Dechaume and Amina Bibi have participated to the SNPs genotyping in the study samples. Guillaume Charpentier, Beverley Balkau and Michel Marre have provided some of the patient and control cohorts included in our study. Fernando Gibson has participated in the discussion of the results and the writing of the paper. Philippe Froguel and Martine Vaxillaire have directed the study and the writing of the paper.

## Pre-publication history

The pre-publication history for this paper can be accessed here:


